# Quantum dynamics study of energy requirement on reactivity for the HBr + OH reaction with a negative-energy barrier

**DOI:** 10.1038/srep40314

**Published:** 2017-01-10

**Authors:** Yuping Wang, Yida Li, Dunyou Wang

**Affiliations:** 1 College of Physics and Electronics, Shandong Normal University, Jinan 250014, Shandong, China

## Abstract

A time-dependent, quantum reaction dynamics approach in full dimensional, six degrees of freedom was carried out to study the energy requirement on reactivity for the HBr + OH reaction with an early, negative energy barrier. The calculation shows both the HBr and OH vibrational excitations enhance the reactivity. However, even this reaction has a negative energy barrier, the calculation shows not all forms of energy are equally effective in promoting the reactivity. On the basis of equal amount of total energy, the vibrational energies of both the HBr and OH are more effective in enhancing the reactivity than the translational energy, whereas the rotational excitations of both the HBr and OH hinder the reactivity. The rate constants were also calculated for the temperature range between 5 to 500 K. The quantal rate constants have a better slope agreement with the experimental data than quasi-classical trajectory results.

The title reaction HBr + OH → Br + H_2_O has attracted great interest with many experimental and theoretical studies during the past several decades. From the practical aspect, this reaction plays an important role in atmospheric chemistry because it produces bromine atoms, and the bromine atoms can very effectively destroy the ozone by a catalytic cycles in the stratosphere: Br + O_3_ → BrO + O_2_, BrO + BrO → 2Br + O_2_. In addition, the reaction also plays a key role in combustion chemistry as some brominated compounds act as fire retardants.

From experimental studies, there is a number of measurements of rate constants mainly at room temperature (298 K)^1^,^2^,^3^,^4^,^5^,^6^. Moreover, the rate constants were also measured at the temperature ranges 249–416^7^, 23–295^8^, 76–242^9^, 230–360^10^, 120–224^11^, 20–350^12^, 53–135 K^13^, and at a high temperature of 1925 K^14^. Among them, four studies^4^,^10^,^11^,^13^ have also measured the rate constants for the isotopomers system and found the primary kinetic isotope effect (KIE) is independent of temperature between 53 and 135 K^13^. The results of these investigations reveal that the HBr + OH reaction’s rate constants are extremely negative temperature-dependent below 150 K and nearly independent temperature between ~400 K and room temperature. Furthermore, Butkovskaya and Setser^15^ studied the vibrational distributions for H_2_O, HOD and D_2_O produced in reactions of OH and OD with HBr and DBr. Che *et al*.^16^ observed the negative collision energy dependence of reaction cross section for the HBr + OH/OD reaction in a crossed molecular beam experiment. Tsai *et al*.^17^,^18^ reported the orientation dependence of the Br formation and found that O-end attack is more favored for this reaction.

From theoretical studies, Clary *et al*.^19^ provided the upper limit of rate constant for HBr + OH at low temperatures and predicted a maximum rate constant with the value of 3.5 × 10^−10^ cm^3^ molecule^−1^ s^−1^ at 20 K, using the statistical adiabatic capture theory with a long-range barrierless electrostatic interaction potential. After that, Clary *et al*.^20^ reported a three-dimensional quantum scattering calculation with the rotating bond approximation on a simple potential energy surface (PES) based on a LEPS function and an accurate H_2_O potential. The reaction cross sections are found to be dependent on (2*j* + 1)^−1^, where *j* is the initial rotational quantum number of OH. And the calculated rate constant has a 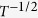 dependence at low temperatures. Furthermore, Nizamov *et al*.^21^ readjusted the LEPS PES^20^ to fit the experimentally measured H_2_O vibrational energy and the thermal rate constant, they performed a quasi-classical trajectory (QCT) study on the mechanism for excitation of the bending mode and isotopic effects on the energy disposal. In 2001, Liu *et al*.^22^ investigated the dynamic properties of the hydrogen abstraction reaction HBr + OH over a wide range of temperatures 23–2000 K, by employing the improved canonical variational transition-state theory (VTST)^23^ with a small-curvature tunneling correction.

Recently, Bowman’s group^24^ has developed a high-quality, full dimensional PES for the HBr + OH system based on 26,000 high-level *ab initio* energies. There is a van der Waals (vdW) well in the entrance channel, as well as in the product channel respectively, and a negative energy saddle-point barrier on the PES. They carried out a QCT calculation to obtain the reaction’s rate constants over the temperature range from 5 to 500 K, and found an inverse temperature dependence of rates below 160 K and a nearly constant temperature dependence above 160 K. In addition, they also studied the reaction cross section, energy disposal and rate constant for the isotopomers reaction DBr + OH^25^. In 2015, Ree *et al*.^26^ reported the temperature dependence of the title reaction using analytic forms of two-, three-, four-body and long-range interaction potentials in a QCT calculation over the temperature range of 20–2000 K. In 2016, Coutinho *et al*.^27^ investigated the stereodirectional dynamics of the title reaction as the prominent reason for the peculiar kinetics on a multidimensional PES mechanically generated on-the-fly^28^.

Till now, there have been no full-dimensional, quantum dynamics studies on the HBr + OH reaction. Thus, in this paper, we carry out the first, full-dimensional, quantum dynamics time-dependent, wave-packet study on the PES developed by Bowman’s group. Our purpose of the present work is to (1) calculate the thermal rate constants over the temperature range of 5–500 K and compare our six degrees of freedom (6DOF) results with experiments^2^,^3^,^4^,^5^,^6^,^7^,^8^,^9^,^10^,^11^,^12^,^13^ and the QCT results^24^, see the relationship of the rate constants with the temperature; (2) investigate the energy efficiency of the translational, vibrational, and rotational energy on a negative-energy barrier.

In recent years, studies on energy efficacy rules for more than three atoms systems show there does not exist a unified rule on the energy efficacy to reactivity regarding the location of the transition states. For example, the O + CH_4_/CD_4_/CHD_3_ reaction with a slightly late barrier, studies^29^,^30^,^31^ on the reactions indicate that the translational energy is more effective than all the vibrational motions in surmounting the slightly late barrier. Similar to the O + CH_4_ reaction, the reaction H + CH_4_ also has a slightly late barrier. However, the quantum dynamics calculations^32^,^33^ show that the vibrational energy is more efficient in promoting the reaction than the translational energy.

The title reaction HBr + OH has a large exoergicity with an early barrier, however, the early barrier is −0.52 kcal mol^−1^ lower than the reactant on the PES. There has been no quantum reaction dynamics studies before on the energy efficacy for the negative early barrier. Since the ground-state energy of the reactant is already higher than the barrier height, there is no barrier for the reactant to surmount, one wonders whether any form of the reactant energy (translational, vibrational or rotational energy) is equal to enhance the reactivity; if not, it would be interesting to find the energy efficacy in surmounting this negative early barrier and to see what energy efficacy rule governs this reaction system.

## Results and Discussion

### Vibrational excitation of HBr

**Figure 1:**
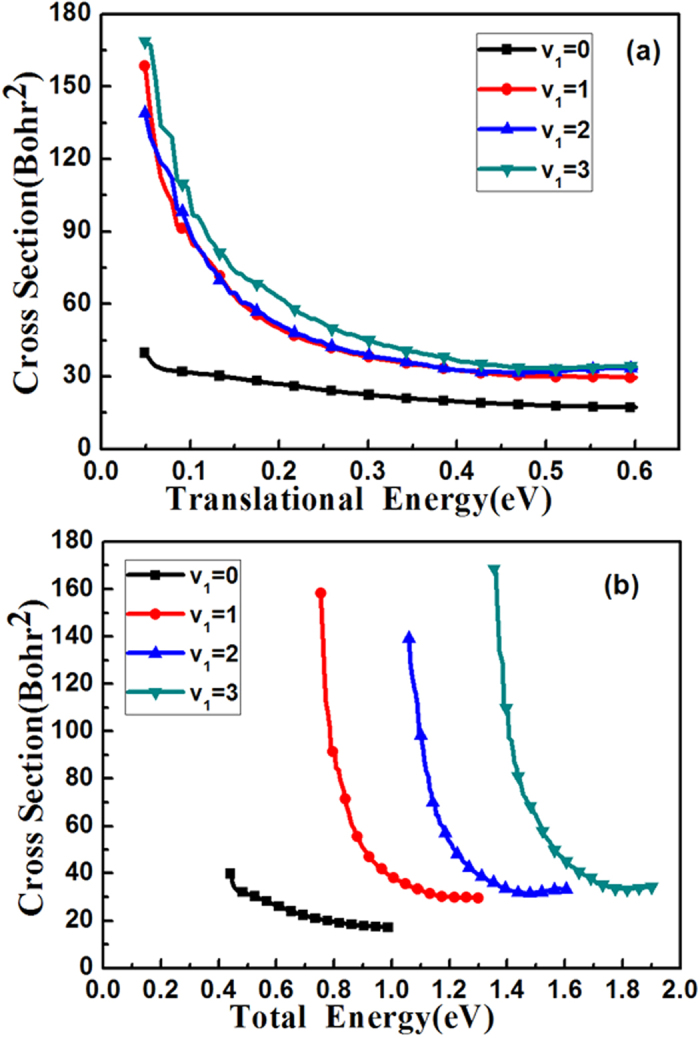
The integral cross sections of the reaction HBr (*v*_1_, *j*_1_ = 0) for *v*_1_ = 0, 1, 2, 3 with OH (*v*_2_ = 0, *j*_2_ = 0) at ground state as a function of translational energy (**a**) and total energy (**b**), respectively.

Figure 1(a) and (b) give the integral cross sections’ (ICSs) comparison for the first four vibrational excitation states of HBr (*v*_1_, *j*_1_ = 0) with OH (*v*_2_ = 0, *j*_2_ = 0) at ground state as a function of translational energy and total energy, respectively. To converge the ICSs for the initial states: (*v*_1_ = 0, *j*_1_ = 0), (*v*_1_ = 1, *j*_1_ = 0), (*v*_1_ = 2, *j*_1_ = 0) and (*v*_1_ = 3, *j*_1_ = 0), 200, 230, 260 and 260 partial waves are calculated, respectively. For the partial waves of *J* ≤ 100, the reaction probability for every partial wave was calculated explicitly, and the reaction probabilities for different partial waves of *J* > 100 were computed using the *J*-shifting method^34^ with a *J* interval of 5. The standard centrifugal sudden (CS) approximation^35^,^36^ was employed in calculation for *J* > 0. This figure shows the cross sections decrease as the translational energy increases. Especially for the excited state ICSs, *v*_1_ = 1, 2, 3, the cross sections decrease significantly about 75% as the translational energy goes from 0.05 to 0.3 eV. On the other hand, the ground state cross section drops slower only about 45% for the same energy. Among these four cross sections, the ground state ICS is the smallest. For the translational energy lower than 0.1 eV, the amplitudes of the HBr *v*_1_ = 1, 2, 3 ICSs are about 3~4 times bigger than that of ground state; even for the collision energy is larger than 0.1 eV, the three excited-state ICSs are also about 2 times bigger than the ground state’s. As this reaction has a negative barrier height, even the ground state energy is higher than the barrier, it is surprising to see that the higher of the excited state the more reactive of this reaction.

**Figure 2:**
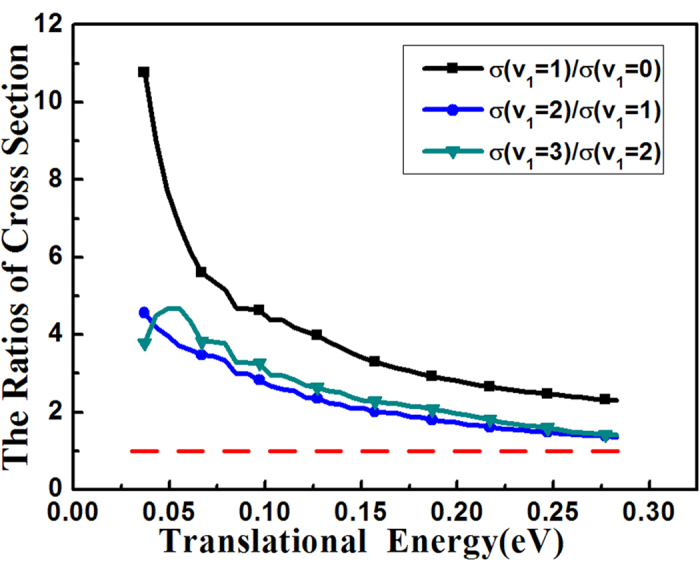
The ICS ratios of *σ(v*_1_ = 1)/*σ(v*_1_ = 0), *σ(v*_1_ = 2)/*σ(v*_1_ = 1) and *σ(v*_1_ = 3)/*σ(v*_1_ = 2) of HBr with OH at ground state as a function of translational energy on the equal amount of total energy.

In order to see the energy efficacy of the vibrational energy of HBr on the reactivity. We plot the ICS ratios of the HBr, *σ(v*_1_ = 1)/*σ(v*_1_ = 0), *σ(v*_1_ = 2)/*σ(v*_1_ = 1) and *σ(v*_1_ = 3)/*σ(v*_1_ = 2), on the basis at an equivalent amount of total energy in Fig. 2. The ICS ratio of *σ(v*_1_ = 1)/*σ(v*_1_ = 0) has a maximum ~10.8 at the initial translational energy 0.037 eV, then rapidly drops to 3.5 at the 0.145 eV until it reaches to 2.2 at 0.283 eV. In the whole energy range, the ratio is always bigger than 1.0, which means that vibrational energy is more effective to promote the reaction than translational energy. Furthermore, the ratio of *σ(v*_1_ = 1)/*σ(v*_1_ = 0) is much bigger than those of *σ(v*_1_ = 2)/*σ(v*_1_ = 1) and *σ(v*_1_ = 3)/*σ(v*_1_ = 2), and the ratio of *σ(v*_1_ = 3)/*σ(v*_1_ = 2) is just slightly larger than *σ(v*_1_ = 2)/*σ(v*_1_ = 1). Bases on the above results, we can conclude that the vibrational excitation from the ground state to the first excited state is the most effective one to promote the reactivity; however, there is no much reactivity change as the vibrational quantum numbers increase from *v*_1_ = 1 to *v*_1_ = 2 and from *v*_1_ = 2 to *v*_1_ = 3. Nevertheless, the comparison of the ICS ratios on the equal amount total energy indicates that the vibrational energy of HBr is more effective than translational energy on promoting the reactivity for this negative-barrier reaction.

### Vibrational excitations of OH

**Figure 3:**
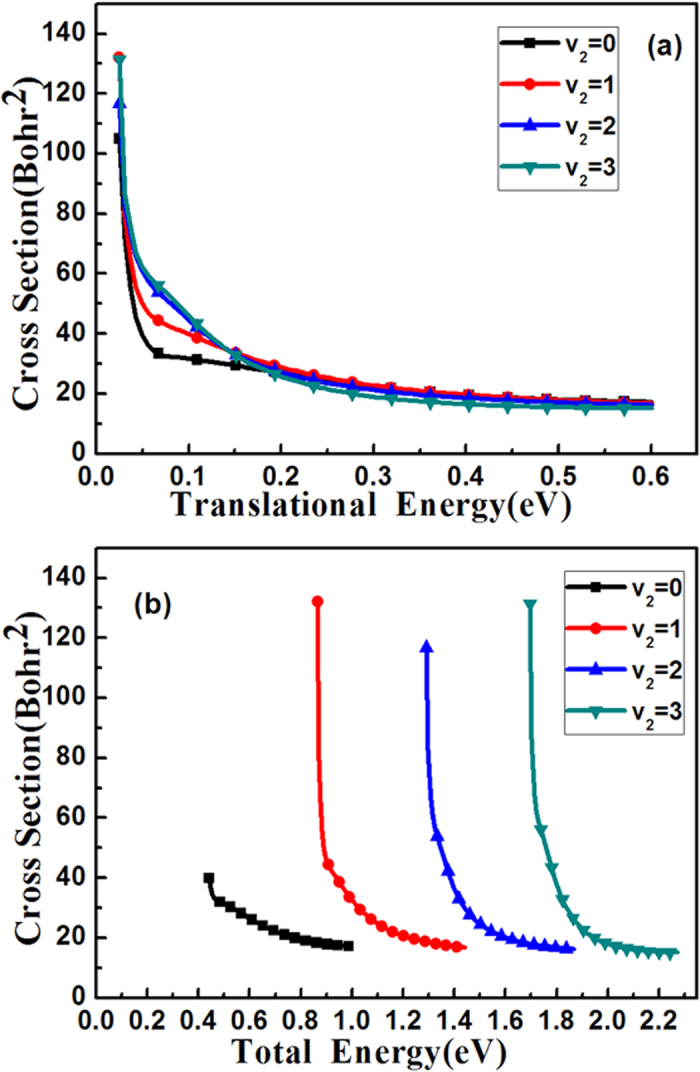
The integral cross sections of the reaction HBr (*v*_1_ = 0, *j*_1_ = 0) at ground state with OH (*v*_2_, *j*_2_ = 0) for *v*_2_ = 0, 1, 2, 3, as a function of translational energy (**a**) and total energy (**b**), respectively.

In Fig. 3(a) and (b), we also compares the ICSs for those vibrational excitation states of OH with HBr (*v*_1_ = 0, *j*_1_ = 0) at ground state as a function of translation energy and total energy, respectively. There are 215, 210 and 210 partial waves needed to converge the vibrational excitation state of OH (*v*_2_ = 1, *j*_2_ = 0), (*v*_2_ = 2, *j*_2_ = 0), (*v*_2_ = 3, *j*_2_ = 0), respectively. The results show that ICSs almost stick together in regard to the translation energy, and the ICSs decrease as the translational energy increases. The reaction path of the PES^24^ we used here has a vdW minimum at the entrance channel with a structure of the O-end of OH linked to HBr, HO
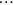
HBr. This means the favorite route of this reaction is the two reactants enter the entrance vdW minimum to form HO
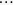
HBr, then scale the transition state to make the reaction occur. This has been confirmed by the crossed beam scattering experiment^17^,^18^ by Tsai *et al*. They found the orientation dependence for the title reaction that the reaction is favored by OH re-orientating its O-end to face the HBr. Since the barrier height respect to the vdW minimum is 0.12 eV on the PES^24^, thus as seen in Fig. 3(a), the reaction ICSs are almost the same at the translational energy larger than ~0.15 eV because the reactants would overpass the vdW minimum without reorientation in this large energy range; however, for the translational energy less than ~0.15 eV, the reactants will enter the vdW minimum to re-orientate themselves then surmount the barrier, therefore the ICSs are bigger for translational energy smaller than 0.15 eV. In order to investigate the vibrational energy efficacy of the OH, we need to check the ICS ratio of the excited state over the ground state in terms of equal amount of the total energy.

**Figure 4:**
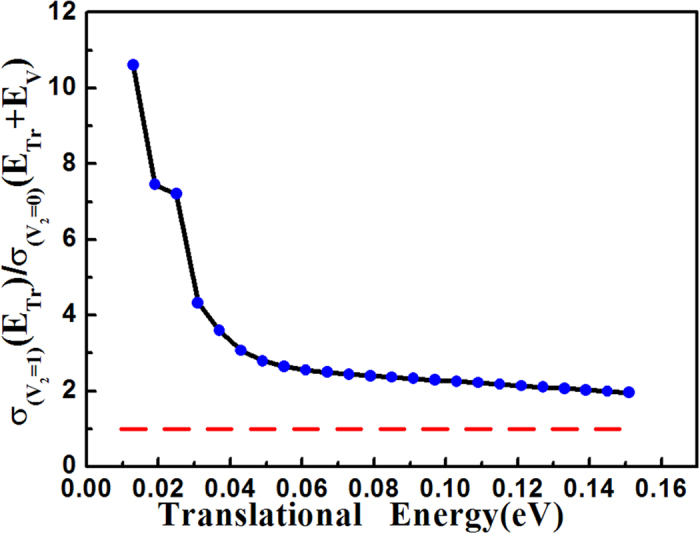
The ICS ratio of OH, *σ(v*_2_ = 1)/*σ(v*_2_ = 0) with HBr at ground state in terms of the translational energy on the basis of equivalent amount of the total energy, where the vibrational energy difference *E*_*V*_ between the two state is 0.448 eV.

Figure 4 gives the ICS ratio, *σ(v*_2_ = 1)/*σ(v*_2_ = 0), in terms of translational energy at the equal amount of total energy. This figure shows that the ratio is larger than 1 in the whole energy range and the ICS ratio curve of *σ(v*_2_ = 1)/*σ(v*_2_ = 0) is very similar to the HBr’s. The ratio is also inversely proportional to the energy and has a rapid decline at lower energy. This shows that the vibrational energy of OH is also much more efficient than the translational energy in promoting the reaction. Compared with the F + CH_4_^37^ and F + H_2_O reaction^38^, the title reaction HBr + OH has some similarities with them in regard to the PES. The three reactions all have an early saddle point located in the reactant channel, a vdW well in the entrance channel, and a relatively deep vdW minimum in the product valley. And the difference is that the HBr + OH reaction has a negative energy barrier (−0.52 kcal mol^−1^) on the PES, while F + CH_4_ barrier height is 0.5 kcal/mol and F + H_2_O’s is 3.8 kcal/mol. For the early barrier reaction HBr + OH, our calculation shows both the vibrational energies of HBr and OH are more effective than translational energy in enhancing the reactivity. While for the two early barrier of F + H_2_O^39^ and F + CHD_3_^40^,^41^ reactions, the study of F + H_2_O shows that the vibrational energy of H_2_O has higher efficacy in enhancing the reactivity than the translational energy; however, the vibrational excitation of C-H stretching motion of CHD_3_ hinders the overall reaction rate. Thus, these investigations further prove that, Polanyi rules^42^ in which the translational energy is more effective to raise the reactivity for the early-barrier tri-atomic reaction systems cannot be extended to the ploy-atomic reaction systems. Nonetheless, this study shows that, for this negative, early barrier reaction, the vibrational energy is more efficient than the translational energy in promoting the reactivity.

### Rotational excitations ICSs

**Figure 5:**
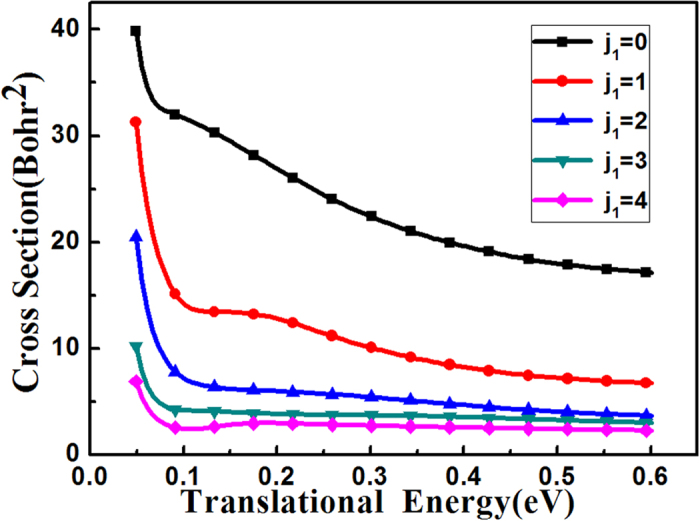
The integral cross sections of the reaction HBr (*v*_1_ = 0, *j*_1_) for *j*_1_ = 0, 1, 2, 3, 4 with OH (*v*_2_ = 0, *j*_2_ = 0) as a function of the translational energy.

In addition, we also studied the rotational excitations on the reactivity for this reaction. For all the excited rotational ICSs’ calculations, 200 partial waves were needed to converge the excited rotational ICSs, and the CS approximation^35^,^36^ was also used to calculate the partial wave reaction probabilities for *J* > 0. Similar to the vibrational ICSs’ calculations, for the reaction probabilities of partial waves for *J* ≤ 100, every *J* partial wave was calculated; and for *J* > 100, the reaction probabilities were computed using the *J*-shifting method^34^ with a *J* interval of 5. Figure 5 presents the first five rotational excited ICSs of the HBr (*v*_1_ = 0, *j*_1_) with OH (*v*_1_ = 0, *j*_1_ = 0) at the ground state as a function of translational energy. As the figure shows that the ground state has the largest ICS among the 5 ICSs, and as the rotational quantum numbers *j*_1_ increases, these excited rotational states’ ICSs significantly decrease. So the rotational-excited modes of the HBr greatly hinder the reactivity.

**Figure 6:**
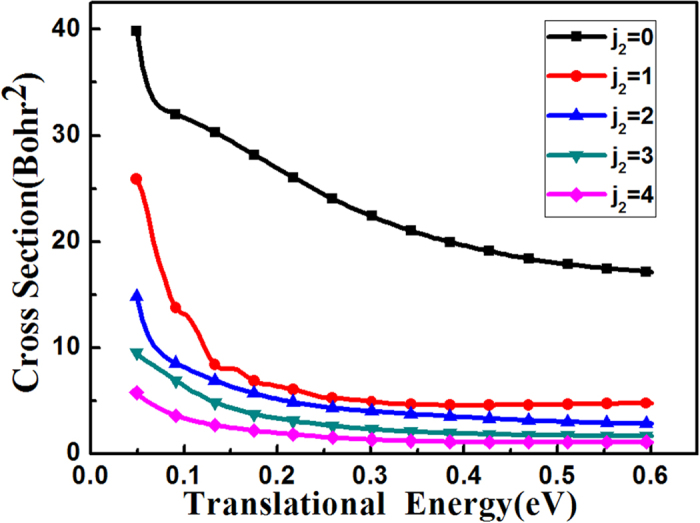
The integral cross sections of the reaction HBr (*v*_1_ = 0, *j*_1_ = 0) with OH (*v*_2_ = 0, *j*_2_) for *j*_2_ = 0, 1, 2, 3, 4 as a function of the translational energy.

In Fig. 6, the first four ICSs of rotational excitations and the ground state of the OH were compared. It is shown that overall the OH rotational excitations greatly inhibit the reactivity, and the faster of the rotation, the smaller of the ICS. This can be explained due to the fact that the OH plays a receiver role, the faster rotation of OH will further add difficulty for H atom in HBr to attack the O atom in OH. And our 6DOF quantum dynamics results here are in agreement with the three-dimensional quantum scattering calculations by Clary *et al*.^20^ who found the reaction cross sections are proportional to (2*j* + 1)^−1^, where *j* is the initial rotational quantum number of OH.

Overall, the rotational excitations, both HBr and OH, hinders the reactivity. This indirectly proves Tsai *et al*. molecular beam study^17^,^18^ that Br formation of this reaction has orientation dependence which favors the O-end attack. On one hand, the faster rotation of HBr will make H in HBr cannot attack O-end easily; on the other hand, the faster rotation of OH will make O-end having difficulty to receive the H in HBr. Therefore, both the rotational excitations of HBr and OH hinder the reactivity.

### Thermal rate constants

By summing over all the ro-vibrational states of HBr and OH, we can obtain the 6DOF cumulative reaction probability (CRP). In order to converge the rate constants up to 500 K to compare with the experimental results^2^,^3^,^4^,^5^,^6^,^7^,^8^,^9^,^10^,^11^,^12^,^13^, the reaction probabilities of the ground vibrational state including 6 HBr rotational excitation states (*j*_1max_ = 5) and 3 OH rotational excitation states (*j*_2max_ = 3) were calculated.

**Figure 7:**
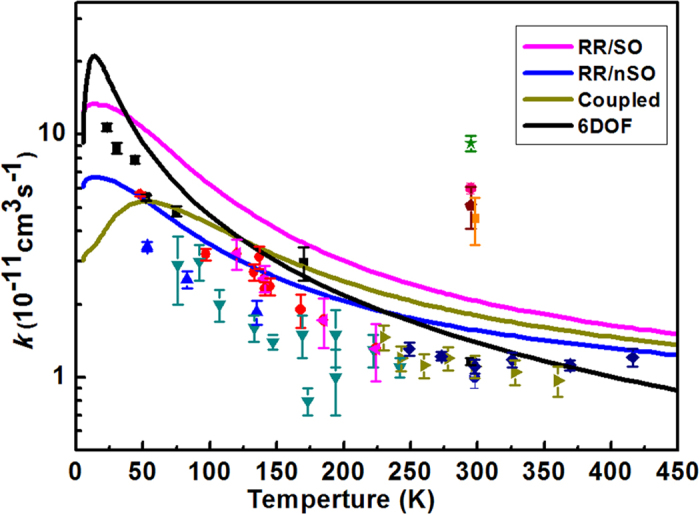
Comparison of the 6DOF thermal rate constants with different experimental data^2^,^3^,^4^,^5^,^6^,^7^,^8^,^9^,^10^,^11^,^12^,^13^ (symbols with error bars) and Bowman’s group QCT results (RR/SO, RR/nSO, Coupled)^14^.

The thermal rate constants are obtained using the *J*-*K* shifting rate expression from equation (8) in the Method Section. Note, in the QCT calculation by Bowman’s group^24^, they compared their calculated rates, with the OH spin-orbit coupling (RR/SO rates), without the spin-orbit coupling(RR/nSO rates), and with a fully coupled partition function (Coupled rates), with the experimental measured ones. They found that, with the spin-orbit coupling neglected, the RR/nSO rates have the best overall agreement with the experimental results. Therefore, in the current study, we neglected the spin-orbit coupling to calculate our 6DOF quantal rate constants. In Fig. 7, our 6DOF results are compared with experimental^2^,^3^,^4^,^5^,^6^,^7^,^8^,^9^,^10^,^11^,^12^,^13^ and QCT results (RR/SO, RR/nSO, Coupled)^24^. And the QCT calculations of RR/SO, RR/nSO and Coupled are obtained from three different approaches to treat the reactant OH rotational and associated electronic partition function. As the comparison shows, our 6DOF rate constants have a good agreement with the experimental data and demonstrate a negative temperature dependence, which is in agreement with the experimental ones and the QCT results. However, at the very low temperature range upto about 50 K, our 6DOF results are bigger than the experimental and QCT results. Nonetheless, in general, our 6DOF results have a better agreement with the slope of the experimental data than the QCT results. In addition, our 6DOF results give a maximum at about 15 K just as RR/SO and RR/nSO results do. This agrees with Clary *et al*.’s^19^ prediction that a maximum rate constant should appear at 20 K. On the whole, Fig. 7 shows that our 6DOF rate constant has a better slope agreement with the experiments than the QCT results except at the extreme low temperature.

## Discussion

In this work, we carried out a 6DOF quantum reaction dynamics, time-dependent wave packet propagation approach to study the HBr + OH → Br + H_2_O reaction system on the PES developed by Bowman’s group. This is the first, full-dimensional, quantum dynamical study on the title reaction. For the HBr + OH reaction system with a negative-early barrier, this study shows that not all forms of energy are equal in enhancing the reactivity. Even the ground state energy of the reactant is higher than the barrier, the calculation still shows that vibrational excitations of both the HBr and OH vibrational enhance the reactivity. Furthermore, the HBr and OH vibrational excitations are more effective in enhancing the reactivity than the translational energy. We also studied the rotational excitations of HBr and OH. The results show that both the rotational excitations hinder the reactivity. This is due to the fact that the faster rotation of HBr makes H having difficult to attack O in OH; and the faster rotation of OH makes O having difficult to receive H from HBr. Comparing with other two early barrier reaction systems, F + H_2_O and F + CHD_3_, we can see there are no general rules so far on the energy efficacy for the ploy-atomic reaction systems as the Polanyi rules do to the tri-atomic systems. We think this is due to the complexity of the PESs of the poly-atomic systems with vdW wells usually in both the reactant and product channels. These wells, especially the entrance channel well before the transition state, might also play a role that cannot be neglected on energy efficacy on surmounting the energy barrier.

Furthermore, the comparison of the thermal rate constants between our 6DOF quantum results and the experimental display that our data agree well with experimental measurement except at extreme low temperatures.

## Theoretical Methods

### 6DOF approach

**Figure 8:**
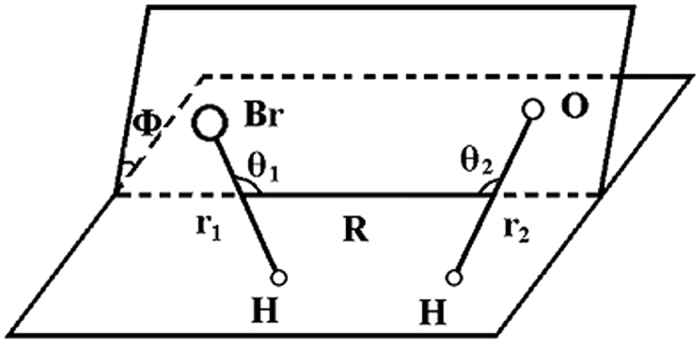
Reactant Jacobi coordinates for the reaction HBr + OH.

We performed a full dimensional, 6DOF, time-dependent quantum dynamics study for the HBr + OH → Br + H_2_O reaction. The 6DOF Hamiltonian in the reactant Jacobi coordinates, as shown in Fig. 8, can be written as,


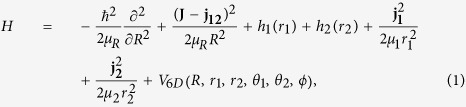


where, *μ*_*R*_ is the reduced mass of the whole reaction system; *R* is the center-of-mass distance between HBr and OH, *r*_1_ is the distance of H-Br and *r*_2_ is the distance of O-H; *θ*_1_ and *θ*_2_ are the two Jacobi angles between *r*_1_ and *R* and *r*_2_ and *R*, Φ is the torsion angle; **J** is the total angular momentum operator of the reaction system, **j**_**1**_ and **j**_**2**_ are the rotational angular momentum operators for HBr and OH, respectively, **j**_**12**_ is the coupled angular momentum operator of **j**_**1**_ and **j**_**2**_; and *V*_6*D*_ is the interaction potential. The vibrational reference Hamiltonians *h*_1_(*r*_1_) and *h*_2_(*r*_2_) are defined as


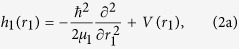



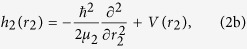


Here *V(r*_1_) and *V(r*_2_) are the one-dimensional reference potentials for HBr and OH, and *μ*_1_ and *μ*_2_ are the corresponding masses. These potentials correspond to the reactant at the asymptotic region with other coordinates fixed at the equilibrium geometry.

The split-operator method^43^ is employed here to propagate the wave packet on the full-dimensional *ab initio* PES for the quantum scattering calculation. And we expand the time-dependent wave-function in terms of the body-fixed (BF) rovibrational eigenfunctions defined in terms of the above reactant Jacobi coordinates. After the time-dependent wave function is propagated into the product region, we perform the standard reactive flux^44^,^45^,^46^,^47^ method to extract the initial-state-selected reaction probability. To obtain the initial-state-selected ICS, we first calculated the partial wave reaction probabilities for the different initial total angular **J**. Then the ICS 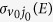 is obtained by summing over all the initial-state-selected reaction probability 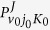 for all partial waves


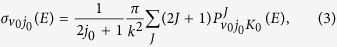


where 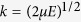 is the wave number and *E* is the translational energy, *v*_0_ and *j*_0_ denotes, respectively, the initial vibrational and rotational quantum numbers, *K*_0_ is the projection of *J* onto BF *z* axis of the reaction system.

### Cumulative reaction probability and thermal rate constant

The CRP *N*^*J*=0^ (*E*) is defined as the sum of all the initial sate selected ro-vibrational reaction probabilities 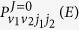



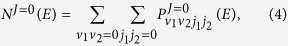


Next, the *J*-shifting method^34^ is employed here to calculate the CRP for the nonzero total angular momentum *J*. The total CRP, *N(E*) is defined as the sum of CRPs for all the open *J* and *K* channels


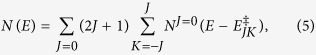


where 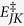 is the rigid rotor rotational energy of the reaction system at the transition state. This energy is approximated by the expression for a symmetric top molecule,


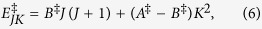


*A*^‡^ and *B*^‡^ are the rotational constants of OHHBr at the transition state.

Thus the thermal rate constant can be computed as


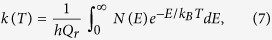


where *Q*_*r*_(*T*) is the reactant partition function, which is written as a product of vibrational, rotational, and translational partition functions. The equation (7) simplifies under the *J*-*K* shifting approximation in terms of Equation (5),


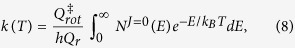


where 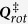 is the rotational partition function of the reaction system at the transition state.

### Numerical aspects

To converge the above 6DOF, wave-packet, quantum dynamics calculation, we used the following numerical parameters to expand the wavefunction: for the translational coordinate *R* from 2.5 to 12.5 bohr, 240 sine basis functions are used, and among these, 150 are used for the interaction region; 30 potential-optimized vibrational discrete variable representation (DVR) points^48^ for the *r*_1_ coordinates in the range from 1.6 to 5.5 bohr; 40 spherical harmonic rotational functions are used for *θ*_1_ and 15 for *θ*_2_, which gives 4896 coupled parity adapted total angular momentum basis. The time-dependent wave packet is propagated for a total time of about 16,000 atomic unit time with a time step of 15 a.u.

For the thermal rate constant calculation in equation (6), the rotational constants *A*^‡^ and *B*^‡^ of OHHBr at the transition state are 15.46 cm^−1^ and 0.14 cm^−1^. For the reactant HBr and OH’s vibrational partition function, the used harmonic vibrational frequencies of HBr and OH are 2525 cm^−1^ and 3611 cm^−1^. For reactant rotational partition function, the HBr’s rotational constant is 8.46 cm^−1^ and the OH’s 18.86 cm^−1^.

## Additional Information

**How to cite this article:** Wang, Y. *et al*. Quantum dynamics study of energy requirement on reactivity for the HBr + OH reaction with a negative-energy barrier. *Sci. Rep.* **7**, 40314; doi: 10.1038/srep40314 (2017).

**Publisher's note:** Springer Nature remains neutral with regard to jurisdictional claims in published maps and institutional affiliations.

## References

[b1] Cannon B. D., Robertshaw J. S., Smith I. W. M. & Williams M. D. A time resolved life study of the kinetics of OH (*v* = 0) and OH (*v* = 1) with HCl and HBr. Chem. Phys. Lett. 105, 380–385 (1984).

[b2] Takacs G. A. & Glass G. P. Reactions of hydrogen atoms and hydroxyl radicals with hydrogen bromide. J. Phys. Chem. 77, 1060–1064 (1973).

[b3] Smith I. W. M. & Zellner R. Rate measurements of reactions of OH by resonance-absorption. 3. Reactions of OH with H_2_, D_2_ and hydrogen and deuterium halides. J. Chem. Soc., Faraday Trans. 2 8, 1045–1056 (1974).

[b4] Husain D., Plane J. M. C. & Slater N. K. H. Kinetic investigation of the reactions of OH (*X*^2^Π) with the hydrogen halides, HCl, DCl, HBr and DBr by time-resolved resonance fluorescence (*A*^2^Σ^+^ − *X*^2^Π). J. Chem. Soc., Faraday Trans. 2 77, 1949–1962 (1981).

[b5] Jourdain J. L., Lebras G. & Combourieu J. Epr determination of absolute rate constants for the reactions of H and OH radicals with hydrogen Bromide. Chem. Phys. Lett. 78, 483–487 (1981).

[b6] Ravishankara A. R., Wine P. H. & Wells J. R. The OH + HBr reaction revisited. J. Chem. Phys. 83, 447–448 (1985).

[b7] Ravishankara A. R., Wine P. H. & Langford A. O. Absolute rate constant for the reaction OH + HBr → H_2_O + Br. Chem. Phys. Lett. 63, 479–484 (1979).

[b8] Sims I. R., Smith I. W. M., Clary D. C., Bocherel P. & Rowe B. R. Ultra-low temperature kinetics of neutral–neutral reactions: new experimental and theoretical results for OH + HBr between 295 and 23 K. J. Chem. Phys. 101, 1748–1751 (1994).

[b9] Atkinson D. B., Jaramillo V. I. & Smith M. A. Low-temperature kinetic behavior of the bimolecular reaction OH + HBr (76–242 K). J. Phys. Chem. A 101, 3356–3359 (1997).

[b10] Bedjanian Y., Riffault V., Le Bras G. & Poulet G. Kinetic study of the reactions of OH and OD with HBr and DBr. J. Photochem. Photobiol., A. 128, 15–25 (1999).

[b11] Jaramillo V. I. & Smith M. A. Temperature-dependent kinetic isotope effects in the gas-phase reaction: OH + HBr. J. Phys. Chem. A. 105, 5854–5859 (2001).

[b12] Jaramillo V. I. . A consensus view of the temperature dependence of the gas phase reaction: OH + HBr → H_2_O + Br. Int. J. Chem. Kinet. 34, 339–344 (2002).

[b13] Mullen C. & Smith M. A. Temperature dependence and kinetic isotope effects for the OH + HBr reaction and H/D isotopic variants at low temperatures (53–135 K) measured using a pulsed supersonic laval nozzle flow reactor. J. Phys. Chem. A. 109, 3893–3902 (2005). 16833707 10.1021/jp045540n

[b14] Wilson W. E., O’Donovan J. T., Fristrom R. M. Flame inhibition by halogen compounds. Symp.(int.) Combust. 12, 929–942 (1969).

[b15] Butkovskaya N. I. & Setser D. W. Chemical dynamics of H abstraction by OH radicals: vibrational excitation of H_2_O, HOD, and D_2_O produced in reactions of OH and OD with HBr and DBr. J. Phys. Chem. 100, 4853–4866 (1996).

[b16] Che D.-C., Matsuo T., Yano Y., Bonnet L. & Kasai T. Negative collision energy dependence of Br formation in the OH + HBr reaction. Phys. Chem. Chem. Phys. 10, 1419–1423 (2008). 18309398 10.1039/b713322g

[b17] Tsai P.-Y., Che D.-C., Nakamura M., Lin K.-C. & Kasai T. Orientation dependence in the four-atom reaction of OH + HBr using the single-state oriented OH radical beam. Phys. Chem. Chem. Phys. 12, 2532–2534 (2010). 20200728 10.1039/b923934k

[b18] Tsai P.-Y., Che D.-C., Nakamura M., Lin K.-C. & Kasai T. Orientation dependence for Br formation in the reaction of oriented OH radical with HBr molecule. Phys. Chem. Chem. Phys. 13, 1419–1423 (2011). 21109858 10.1039/c0cp01089h

[b19] Clary D. C., Stoecklin T. S. & Wickham A. G. Rate constants for chemical reactions of radicals at low temperatures. J. Chem. Soc., Faraday Trans. 89, 2185–2191 (1993).

[b20] Clary D. C., Nyman G. & Hernandez R. Mode selective chemistry in the reactions of OH with HBr and HCl. J. Chem. Phys. 101, 3704–3714 (1994).

[b21] Nizamov B., Setser D. W., Wang H. B., Peslherbe G. H. & Hase W. L. Quasiclassical trajectory calculations for the OH (*X*^2^Π) and OD (*X*^2^Π) + HBr reactions: energy partitioning and rate constants. J. Chem. Phys. 105, 9897–9911 (1996).

[b22] Liu J. Y., Li Z. S., Dai Z. W., Huang X. R. & Sun C. C. Direct *ab initio* dynamics calculations of the reaction rates for the hydrogen abstraction OH + HBr → H_2_O + Br. J. Phys. Chem. A. 105, 7707–7712 (2001).

[b23] Truhlar D. G. & Garrett B. C. Variational transition-state. Acc. Chem. Res. 13, 440–448 (1980).

[b24] de Oliveira-Filho A. G. S., Ornellas F. R. & Bowman J. M. Quasiclassical trajectory calculations of the rate constant of the OH + HBr → Br + H_2_O reaction using a full-dimensional *ab initio* potential energy surface over the temperature range 5 to 500 K. J. Phys. Chem. Lett. 5, 706–712 (2014). 26270841 10.1021/jz5000325

[b25] de Oliveira-Filho A. G. S., Ornellas F. R. & Bowman J. M. Energy disposal and thermal rate constants for the OH + HBr and OH + DBr reactions: quasiclassical trajectory calculations on an accurate potential energy surface. J. Phys. Chem. A. 118, 12080–12088 (2014). 25365787 10.1021/jp509430p

[b26] Ree J., Kim Y. H. & Shin H. K. Dependence of the four-atom reaction HBr + OH → Br + H_2_O on temperatures between 20 and 2000 K. J. Phys. Chem. A. 119, 3147–3160 (2015). 25751761 10.1021/jp511505h

[b27] Coutinho N. D. . Stereodirectional origin of anti-arrhenius kinetics for a tetraatomic hydrogen exchange reaction: Born–Oppenheimer molecular dynamics for OH + HBr. J. Phys. Chem. A. 120, 5408–5417 (2016). 27205872 10.1021/acs.jpca.6b03958

[b28] Coutinho N. D. . Stereodynamical origin of anti-arrhenius kinetics: negative activation energy and roaming for a four-atom reaction. J. Phys. Chem. Lett. 6, 1553–1558 (2015). 26263312 10.1021/acs.jpclett.5b00384

[b29] Liu R. . Mode selectivity for a “central” barrier reaction: eight-dimensional quantum studies of the O (^3^P) + CH_4_ → OH + CH_3_ reaction on an *ab initio* potential energy surface. J. Phys. Chem. Lett. 3, 3776–3780 (2012). 26291110 10.1021/jz301735m

[b30] Czakó G. & Bowman J. M. Dynamics of the O (^3^P) + CHD_3_ (*v*_*CH*_ = 0, 1) reactions on an accurate *ab initio* potential energy surface. Proc. Natl. Acad. Sci. USA 109, 7997–8001 (2012). 22566657 10.1073/pnas.1202307109 PMC3361422

[b31] Yan W., Meng F. B. & Wang D. Y. Quantum dynamics study of vibrational excitation effects and energy requirement on reactivity for the O + CD_4_/CHD_3_ → OD/OH + CD_3_ reactions. J. Phys. Chem. A. 117, 12236 (2013). 24152064 10.1021/jp4090298

[b32] Welsch R. & Manthe U. Communication: ro-vibrational control of chemical reactivity in H + CH_4_ → H_2_ + CH_3_: full dimensional quantum dynamics calculations and a sudden model. J. Chem. Phys. 141, 051102 (2014). 25106559 10.1063/1.4891917

[b33] Welsch. R. & Manthe U. The role of the transition state in polyatomic reactions: initial state-selected reaction probabilities of the H + CH_4_ → H_2_ + CH_3_ reaction. J. Chem. Phys. 141, 174313 (2014). 25381520 10.1063/1.4900735

[b34] Bowman J. M. Reduced dimensionality theory of quantum reactive scattering. J. Phys. Chem. 95, 4960 (1991).

[b35] Tack P. R. Space-fixed vs body-fixed axes in atom-diatomic molecule scattering. Sudden approximations. J. Chem. Phys. 60, 633 (1974).

[b36] McGuire P. & Kouri D. J. Quantum mechanical close coupling approach to molecular collisions. jz-conserving coupled states approximation. J. Chem. Phys. 60, 2488 (1974).

[b37] Czakó G., Shepler B. C., Braams B. J. & Bowman J. M. Accurate *ab initio* potential energy surface, dynamics, and thermochemistry of the F + CH_4_ → HF + CH_3_ reaction. J. Chem. Phys. 130, 084301 (2009). 19256605 10.1063/1.3068528

[b38] Li J., Dawes R. & Guo H. An *ab initio* based full-dimensional global potential energy surface for FH_2_O (*X*^2^*A*) and dynamics for the F + H_2_O → HF + HO reaction. J. Chem. Phys. 137, 094304 (2012). 22957566 10.1063/1.4748857

[b39] Li J., Jiang B. & Guo H. Reactant vibrational excitations are more effective than translational energy in promoting an early-barrier reaction F + H_2_O → HF + OH. J. Am. Chem. Soc. 135, 982–985 (2013). 23301908 10.1021/ja311159j

[b40] Zhang W., Kawamata H. & Liu K. CH stretching excitation in the early barrier F + CHD_3_ reaction inhibits CH bond cleavage. Science. 325, 303–306 (2009). 19608914 10.1126/science.1175018

[b41] Czako G. & Bowman J. M. CH stretching excitation steers the F atom to the CD bond in the F + CHD_3_ reaction. J. Am. Chem. Soc. 131, 17534–17535 (2009). 19908862 10.1021/ja906886z

[b42] Polanyi J. C. Some Concepts in Reaction Dynamics. Science 236, 680–690 (1987). 17748308 10.1126/science.236.4802.680

[b43] Fleck J. A. Jr., Morris J. R. & Feit J. R. Time-dependent propagation of high energy laser beams through the atmosphere. Appl. Phys. 10, 129–160 (1976).

[b44] Zhang D. H. & Zhang J. H. Z. Full-dimensional time-dependent treatment for diatomdiatom reactions: The H_2_ + OH reaction. J. Chem. Phys. 101, 1146–1156 (1994).

[b45] Zhang D. H. & Zhang J. H. Z. Quantum reactive scattering with a deep well: time-dependent calculation for H + O_2_ reaction and bound state characterization for HO_2_. J. Chem. Phys. 101, 3671–3678 (1994).

[b46] Wang D. Y. An eight-degree-of-freedom quantum dynamics study for the H_2_ + C_2_H system. J. Chem. Phys. 123, 194302 (2005). 16321083 10.1063/1.2122707

[b47] Wang D. Y. & Huo W. M. An eight-degree-of-freedom quantum dynamics study of the isotopic effect on the reaction HD + C_2_H. J. Chem. Phys. 129, 084303 (2008). 19044819 10.1063/1.2971184

[b48] Echave J. & Clary D. C. Potential optimized discrete variable representation. Chem. Phys. Lett. 190, 225–230 (1992).

